# Mesoporous Silica Nanoparticle–Based Combination of NQO1 Inhibitor and 5-Fluorouracil for Potent Antitumor Effect Against Head and Neck Squamous Cell Carcinoma (HNSCC)

**DOI:** 10.1186/s11671-019-3224-3

**Published:** 2019-12-19

**Authors:** Jing Chen, Shuzhen Zhang, Shuai Zhang, Shanjun Gao, Jianbo Wang, Dongchun Lei, Pengqiang Du, Zhiwei Xu, Cailiang Zhu, Hongbin Sun

**Affiliations:** 1 Department of Dermatology, Henan Provincial People’s Hospital, Zhengzhou University People’s Hospital, Henan University People’s Hospital, Zhengzhou, Henan China; 2 Microbiome Laboratory, Henan Provincial People’s Hospital, Zhengzhou University People’s Hospital, Zhengzhou, Henan China; 3 Department of Pharmacy, Henan Provincial People’s Hospital, Zhengzhou, Henan China; 4 Department of Pharmacy of Central China Fuwai Hospital, Zhengzhou University, Zhengzhou, Henan China; 5 Clinical Research Service Center, Henan Provincial People’s Hospital, Zhengzhou University People’s Hospital, Henan University People’s Hospital, Zhengzhou, Henan China; 6 School of Food and Biological Engineering, Zhengzhou University of Light Industry, Zhengzhou, Henan China

**Keywords:** Squamous cell carcinoma, NQO1 inhibitor, Lipid bilayer, Mesoporous silica nanoparticles, Apoptosis

## Abstract

Head and neck squamous cell carcinomas (HNSCC) are one of the deadliest forms of cancer, and 90% of its origin is from squamous cells. NAD(P)H:quinone oxidoreductase 1 (NQO1), an enzyme overexpressed in squamous cell carcinoma, plays an important role in proliferation and chemoresistance. The main aims were to study the inhibitory effect of ß-lapachone (ARQ761 in clinical form) in HNSCC and to study the combinational effect of 5-FU and ß-lap in improving the therapeutic efficacy in HNSCC. Lipid bilayer–assembled mesoporous silica nanoparticles loaded with 5-FU/ß-lap were prepared and studied for its physicochemical and biological properties. ß-lap showed a concentration-dependent inhibition of NQO1 enzyme activity in Cal33 cells. Notably, significant inhibitory effect was observed at a dose of 20–50 μg/ml of ß-lap. Combination of 5-FU+ß-lap resulted in lower cell viability; most notably, 5-FU/ß-lap-loaded mesoporous silica nanoparticles (FNQ-MSN) exhibited significantly lower cell viability compared with that of any of the individual drug or physical combinations. ß-lap resulted in a decrease in the protein band of NQO1 compared with control; however, most notable decrease in the NQO1 level was observed in the FNQ-MSN-treated cell group. FNQ-MSN resulted in more than 60% of cell apoptosis (early and late apoptosis) and predominant nuclear fragmentation of cancer cells indicating the superior anticancer effect of a carrier-based combination regimen. Notable decrease in tumor volume was observed with the physical mixture of 5-FU+ß-lap; however, combined treatment of carrier-based 5-FU and ß-lap (FNQ-MSN) significantly delayed the tumor growth and prolonged the survival of tumor-bearing xenograft mice. These findings suggest the potential of NQO1 inhibitor in enhancing the chemotherapeutic potential of 5-FU in the treatment of HNSCC.

## Introduction

Head and neck squamous cell carcinomas (HNSCC) are one of the deadliest forms of cancer and 90% of its origin is from squamous cells [1]. HNSCC is highly angiogenic in nature and its vasculature expresses various cytokines including fibroblast growth factors (FGFs) and vascular endothelial growth factors (VEGFs) which are linked with higher metastasis and poor survival [2]. The incidence of HNSCC in China is sixth in cancer-related death with approximately 250,000 new cases were registered in 2015 and 77,500 cases of death. The overall 5-year survival rate of HNSCC is very low due to the factors including aggressive nature, early relapse, high metastasis, and high mortality rate due to poor prognosis [3]. The main treatment option in HNSCC is surgical procedure followed by radiotherapy or chemotherapy. To be specific, chemotherapy if used effectively at early stages or at post-surgical stages would counteract tumor growth [4, 5]. Effective use of chemotherapy will eradicate the cancer cells within the tumor tissues and inhibit the tumor relapse. However, long-term chemotherapy based on single agent often results in drug resistance and reduced therapeutic efficacy [6]. Therefore, it is necessary to employ innovative strategies to improve the survival and quality of life of HNSCC patients.

Several anticancer agents used on the treatment of HNSCC include cisplatin, 5-fluorouracil (5-FU), paclitaxel, or docetaxel. Among all, 5-fluorouracil (5-FU) is employed as first-line therapy in HNSCC and other squamous cell carcinoma treatment [7]. 5-FU is a pyrimidine analog that acts by irreversibly inhibiting the thymidylate synthase within the cancer cells and thereby suppresses the replication of DNA resulting in cell death [8]. As with all anticancer drugs, 5-FU is not without adverse effect in the systemic circulation and causes toxicity to the normal tissues, while it is reported that the combination of 5-FU with a secondary agent could potentially reduce the side effects and improve the overall therapeutic efficacy in cancer treatment [9].

NAD(P)H:quinone oxidoreductase 1 (NQO1) is a flavoprotein that is elevated in the tumor tissues in the range of 100-200-fold compared with that of normal tissues [10]. The two-electron oxidoreductase is an inducible phase II detoxifying enzyme capable of detoxifying quinones by forming stable hydroquinones [11]. NQO1 is shown to express in a low basal level in normal human tissues where it protects the cells against the redox cycling and oxidative stress and stabilized the p53 suppressor. NQO1 is constitutively overexpressed in several cancers including breast, pancreatic, and squamous cell carcinomas [12]. The overexpression of NQO1 promotes the progression of cancer burden and makes the cancer cells more resistant to chemotherapeutic drugs such as 5-FU or cisplatin (oxidative stress inducers) making NQO1 as a potential oncotarget to improve the therapeutic efficacy [13]. It has been reported that the knockdown of NQO1 by siRNA enhanced the cytotoxic effect of multiple drugs like gemcitabine or doxorubicin [14]. Besides, several synthetic and natural NQO1 inhibitors are reported such as coumarins or curcumins or ES936 [15–17]. In this study, we have employed ß-lapachone (ß-lap, 3,4-Dihydro-2,2-dimethyl-2H-naphtho [1,2-b]pyran-5,6-dione) as a NQO1 inhibitor [18]. ß-lap acts through an NQO1-dependent mechanism and creates a significant amount of reactive oxygen species (ROS) which will lead to damage to the DNA strands and cause cancer cell death [19].

Employing nanoparticles for the systemic delivery of small molecules becomes a promising approach in cancer treatment [20]. The drug-loaded nanoparticles require less dosing schedule and shown to improve the anticancer efficacy and relatively reduce the adverse effects. Among all carriers, mesoporous silica nanoparticle (MSN) holds significant potential to effectively deliver the payload to the tumor tissues [21, 22]. The micrometer-sized pores allow the stable loading of the drugs and prevent its release in the systemic circulation and allow the preferential accumulation in the leaky cancer tissues using enhanced permeation and retention (EPR) effect [23].

Overall, the main aim of the present study was to combine the therapeutic benefits of 5-FU and NQO1 inhibitor to enhance the anticancer effect in HNSCC. The in vitro anticancer effect was analyzed using various techniques such as cell viability, western blot analysis, flow cytometer/Hoechst-based apoptosis assay, and live/dead assay. In vivo studies were performed on the Cal33 tumor cell bearing xenograft model.

## Conclusion

In summary, we have successfully formulated a 5-FU+ß-lap-loaded lipid bilayer–coated mesoporous silica nanoparticles. We have showed that (i) antitumor effect of ß-lap in a concentration-dependent manner in HNSCC tumor cells, (ii) combination of 5-FU+ß-lap results in significant inhibition of NQO1 protein and Bcl-2 protein, (iii) combination-based FNQ-MSN demonstrated a significant reduction in the tumor burden in HNSCC xenograft. These data unequivocally point to the fact that sublethal dose of NQO1 inhibitor (ß-lap) could potentially enhance the therapeutic efficacy of 5-FU in HNSCC tumors and could potentially be extended to the clinical treatment of other malignancies.

## Materials and Methods

### Preparation of 5-FU/ß-lap-Loaded Lipid Bilayer-Mesoporous Silica Nanoparticles

The drug-loaded MSN was prepared by dissolving cetyltrimethylammonium bromide (CTAB) (210 mg) in 180 ml of water and boiled to 80 °C, followed by 5-FU and ß-lap were added 20% w/w of total nanoparticles and then ammonium fluoride (NH4F) (30 mg) was added and stirred well for 60 min. Next, 1.6 ml of tetraethyl orthosilicate (TEOS) was added in a dropwise manner for 30 min and continuously stirred for 3 h. Semi-transparent white colloids were formed which were collected after centrifuging (15 min) at 10000 rpm at 24 ± 1 °C. The MSN were resuspended in ultrapure water, and centrifugation cycle was repeated 2 times. Separately, a thin-film membrane was prepared using DSPE-PEG2000 (2% of total MSN weight) and hydrated with water suspended with drug-loaded MSN. The mixture was immediately probe sonicated for 5 min at 50 W at room temperature (25 °C). The PEGylated lipid bilayer supported MSN was finally washed two times and resuspended in ultrapure water.

### Particle Size, Zeta Potential Analysis, and Morphology Analysis

The particle diameter, polydispersity index (PDI), and zeta potential were evaluated using Zetasizer Nano ZS (Malvern Instruments, Malvern, UK). The particles were analyzed using dynamic light scattering (DLS) mechanism and particle dispersions were well diluted before the experiment. All experiments were performed in triplicate at room temperature. The morphology of nanoparticles was determined by transmission electron microscopy (TEM) using CM 200 UT, Philips, MA, USA) operated at 100 kV. Briefly, particle dispersions were diluted a drop was placed in a 300-mesh TEM grid and allowed to settle for 10 min. The particles were counterstained with 2% phosphotungstic acid (PTA) as negative staining, air-dried, and observed under a TEM microscope.

### Drug Loading

Two ways drug loading analysis was performed by calculating the unloaded or unbound drug in the supernatant and drug loaded in the nanoparticle. The drug loading efficiency was performed by HPLC method. The HPLC was equipped with Shimadzu LC-20 AD PLC pump and SPD-M20A PDA detector and analysis software Shimadzu LC containing reverse phase C18 column (Phenomenex C18, 150 4.6 mm, 5 μm). The mobile phase for 5-FU consists of a mixture of acetonitrile and water (10:90, v/v) and pumped at a rate of 1 ml/min. The detection wavelength set at 265 nm for 5-FU. The mobile phase for ß-lap consisting of acetonitrile/water (31:69, v/v) and detection wavelength set at 254 nm at 35 °C. The loading capacity (LC) and loading efficiency (LE) of 5-FU/ ß-lap were calculated using the respective formula.

$$ \mathrm{LE}\ \left(\%\right)={W}_{t\mathrm{otal}\ 5-\mathrm{FU}+\mathrm{\ss}-\mathrm{lap}}-{W}_{\mathrm{free}\ 5-\mathrm{FU}+\mathrm{\ss}-\mathrm{lap}}/{W}_{\mathrm{total}\ 5-\mathrm{FU}+\mathrm{\ss}-\mathrm{lap}}\times 100 $$

$$ \mathrm{LC}\ \left(\%\right)={W}_{\mathrm{total}\ 5-\mathrm{FU}+\mathrm{\ss}-\mathrm{lap}}-{W}_{\mathrm{free}\ 5-\mathrm{FU}+\mathrm{\ss}-\mathrm{lap}}/{W}_{\mathrm{total}\ \mathrm{NP}\ \mathrm{mass}}\times 100 $$

### Drug Release Study

The release of 5-FU and ß-lap was evaluated in PBS (pH 7.4) and ABS (pH 5.0) at 37 °C using the dialysis method. Briefly, 1 mg equivalent of each drug-loaded nanoparticle was loaded in a dialysis membrane in 1 ml of respective buffer and ends are sealed. The sealed dialysis membrane was placed in 25 ml of respective ABS and PBS buffer and placed in a shaking water bath at 37 °C. Samples were collected at predetermined time interval and replaced with an equal volume of fresh buffer. The amount of drug released in the respective buffer was evaluated by the HPLC method as described in the earlier section.

### Cell Culture and NAD(P)H:Quinone Oxidoreductase 1 (NQO1) Activity Assay

The Cal33 HNSCC cell was cultured in Dulbecco’s modified Eagle’s medium (DMEM) supplemented with 10% of FBS and 1% of antibiotic mixture. The cells were maintained in ambient conditions in an incubator. For NQO1 activity assay, Cal33 cells were seeded in 96-well plate at a seeding density of 8 × 10^3^ cells per well and incubated overnight. The cells were treated with different concentrations of ß-lap and incubated for 24 h. 0.8% digitonin (50 μl of 2 nM EDTA) was used to lyse the cells and the assay was performed using menadiol and MTT (3-(4,5-dimethylthiazol-2-yl)-2,5-diphenyltetrazolium bromide) as a substrate coupling reaction and activity was measured at 620 nm. The assay was performed in triplicate.

### In Vitro Cell Viability Assay

Cell viability assay of ß-lap in increasing concentration and cell viability assay of individual and combinational regimen were tested by a 3-(4,5-dimethylthiazol-2-yl)-5-(3-car boxymethoxyphenyl)-2-(4-sulfophenyl)-2H-tetrazolium (MTS) assay. Briefly, Cal33 cells were seeded in a 96-well plate at a seeding density of 1 × 10^4^ cells per well and incubated overnight. Next day, cells were treated with an increasing concentration of ß-lap, separately; cells were treated with individual and combinational regimen of 5-FU and ß-lap at a base concentration of 5, 10, and 20 μg/ml and incubated for 24 h. The cells were washed twice and treated with MTS solution as per the manufacturer’s guidelines. The cell viability was calculated with regard to untreated cells, and a microplate reader was used for absorbance at 460 nm.

### Western Blot Analysis

The seeded cells in a 6-well plate were treated with increasing concentration of ß-lap, separately; cells were treated with individual (5-FU or ß-lap) and combinational regimen of 5-FU and ß-lap (FNQ-MSN) and incubated for 24 h. The cells were harvested, lysed (M-per buffer), and centrifuged, and the supernatant containing protein was collected. The protein concentration in the cell lysate was evaluated using the bicinchoninic acid (BCA) method. 10% of Bis-Tris polyacrylamide gel was used to separate the proteins and immediately transferred to a polyvinylidene fluoride (PVDF) membrane. The membrane was blocked using 5% skim milk prepared in n Tris-buffered saline (TBS) buffer containing Tween 20 (TBST, pH 7.2). Primary antibodies of rabbit polyclonal NQO1 (1:1000), mouse monoclonal Bcl-2 (1:1000), and mouse monoclonal GAPDH (1:1000) were incubated on membrane overnight at 4 °C. The membrane was washed with TBST and then incubated with secondary antibodies (antimouse or antirabbit IgG) at a dilution of 1:10000 dilutions. The membrane was again washed with TBST and blots were exposed to ECL substrate solution and densities of the protein bands were evaluated using photodeveloper.

### Apoptosis and Hoechst Assay

Cal33 cells were seeded in a 6-well plate at a seeding density of 2 × 10^5^ cells per well and incubated overnight. The cells were then treated with individual (5-FU or ß-lap) and combinational regimen of 5-FU and ß-lap (FNQ-MSN) and incubated for 24 h. The cells were harvested, washed, centrifuged, and pelleted. The cells were treated with 2.5 μl of annexin V and 2.5 μl of PI and incubated for 15 min in dark conditions. The cells were then made up to 1 ml and evaluated using fluorescence-activated cell sorting (FACS, BD, FACSverse). A total of 10,000 cells were analyzed. Similar procedure for cell seeding and drug treatment was followed and then stained with Hoechst 33342 dye (10 μg/ml) and incubated for 10 min. The cells were washed and fixed with 4% paraformaldehyde (PFA) and washed again. The cell morphology was observed under a fluorescence microscope (Nikon A1, Japan).

### Antitumor Efficacy of FNQ-MSN IN HNSCC Xenograft Tumor Model

Eighteen- to twenty-two-gram male BALB/c mice averaging 4–5-week old were procured from the In-House Animal Facility Center of Zhengzhou University of Light Industry, Henan. The animals were maintained in an air-conditioned room with a 12 h dark-light cycle and given free access to food and water. All animal protocols were approved by the Ethics Committee of Zhengzhou University of Light Industry, Henan. To establish a xenograft model, 1 × 10^7^ Cal33 cells in 150 μl of culture media were inoculated in the right hip of the mouse subcutaneously. When the average size of tumor reaches 80–100 mm3, mice were randomly divided into 5 groups for control, 5-FU, ß-lap, 5-FU+ß-lap, and FNQ-MSN, respectively with 8 mice in each group. The 5-FU was administered at a fixed dose of 5 mg/kg and ß-lap at a fixed dose of 25 mg/kg and administered 3 times with a gap of 3 days for every tail vein injection. Mouse weight and tumor volume were regularly monitored for 18 days. The tumor volume was calculated using the formula

$$ V\ \left({\mathrm{mm}}^3\right)={\mathrm{Width}}^2\ \left({\mathrm{mm}}^2\right)\times \mathrm{Length}\ \left(\mathrm{mm}\right)/2 $$

The present study did not observe any mouse death due to tumor cell loading and all animals were euthanized with CO_2_ and then sacrificed by cervical dislocation towards the end of the study.

### Statistical Analysis

Multiple groups were compared using two-way analysis of variance (ANOVA). Significance level of *p* < 0.05 was considered significant and all data are presented as a mean ± standard deviation unless and otherwise it is specifically mentioned in the respective experiments.

## Result and Discussion

### Preparation of 5-FU/ß-lap-Loaded Lipid Bilayer Supported Mesoporous Silica Nanoparticles

**Fig. 1:**
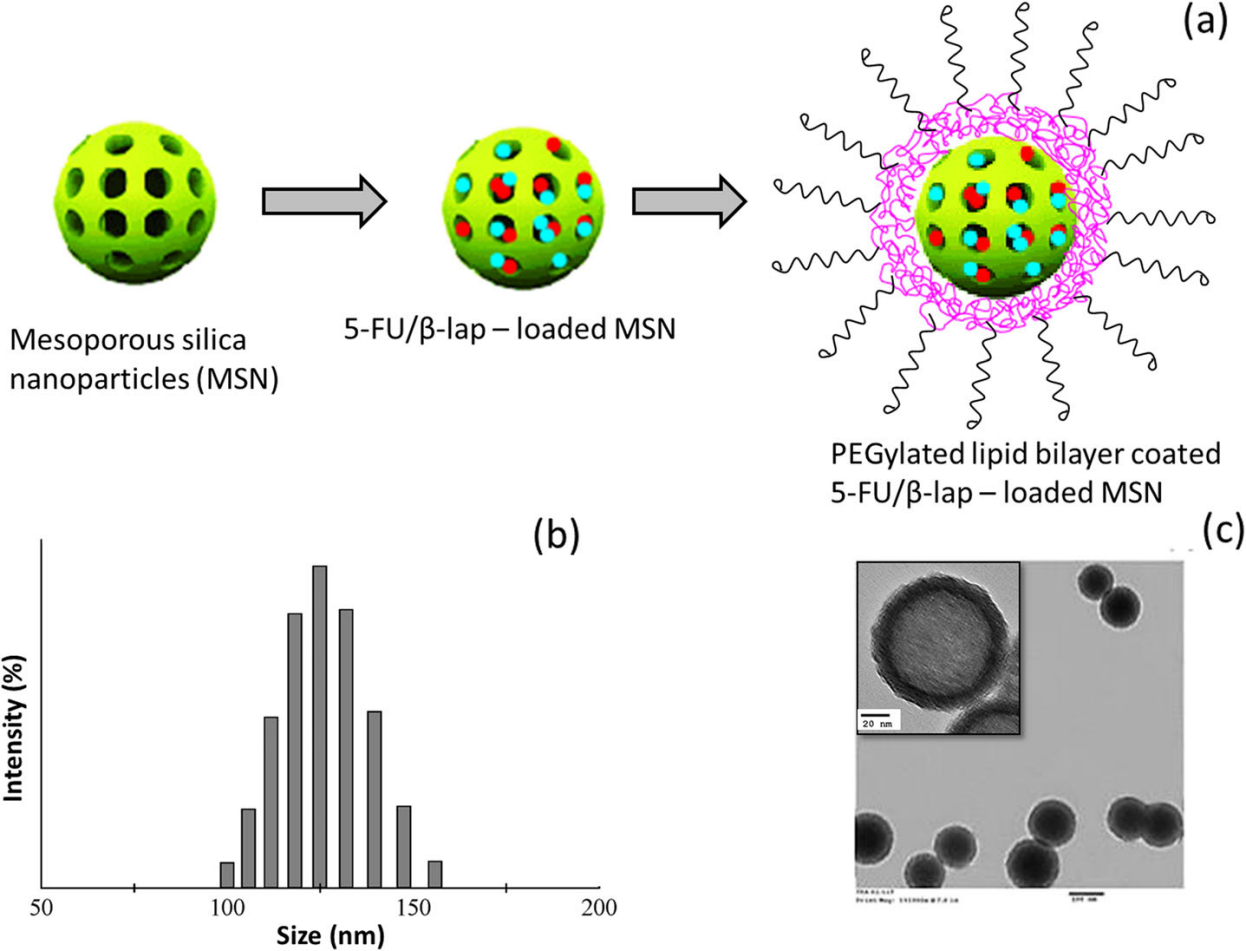
**a** Schematic presentation of the construction of 5-FU and ß-lap-loaded lipid bilayer–coated mesoporous silica nanoparticles. Two drugs are loaded in the pores of the MSN which were further stabilized by the PEGylated lipid bilayer assembly on the outer surface of MSN. **b** Particle size distribution of FNQ-MSN. **c** TEM image of FNQ-MSN with a inset of higher magnifications

In this study, we have customized a mesoporous silica nanoparticle stabilized by PEGylated lipid bilayer. The 5-FU/ß-lap-loaded MSN was prepared by the emulsion-sonication method, and then drug-loaded MSN was introduced in the flask containing lipid thin film consisting of DSPE-PEG2000. The mixture was sonicated and the lipid-coated MSN containing 5-FU/ß-lap was obtained (FNQ-MSN) (Fig. 1a). Loading efficiencies (LE) of 5-FU and ß-lap were 91.5 ± 1.65% and 92.9 ± 1.24%, respectively. The FNQ-MSN exhibited an 8.1 ± 1.12 wt% of 5-FU and 7.6 ± 0.95 wt% of ß-lap indicating the high loading capacity (LC) of MSN carrier. The presence of lipid bilayer will prevent its unwanted release in the systemic circulation and thereby prevents the toxicity. The average particle size of drug-loaded MSN was 92.1 ± 0.85 nm (PDI ~ 0.089) while assembly of lipid bilayer on the MSN surface (FNQ-MSN) increased the average particle size to 128.4 ± 1.24 nm (PDI ~ 0.115) indicating a solid presence of lipid materials on the surface of MSN (Fig. 1b). The zeta potential changed from − 18.2 ± 1.22 to − 26.4 ± mV. The small particle size of FNQ-MSN will allow the preferential accumulation of drug-loaded carrier in the leaky tumor tissues by virtue of the EPR effect. Besides, surface PEGylated will confer the excellent stability and prolonged blood circulation time that will further increase the chances of FNQ-MSN in the tumor tissues. TEM image showed a morphological resemblance to that of liposome and presented a perfect spherical shaped uniformly spread on the grid. TEM image clearly showed a greyish outer later and darker core indicating the presence of lipid assembly on the MSN surface (Fig. 1c). The size observed from TEM corroborates with the DLS particle size from a zetasizer. MSN possess well-ordered channels for the homogeneous distribution of drug molecules. The surface property of pores is one of the important factors in the stable loading of the small molecules. The various physical forces involved in the host-guest interaction mainly include the hydrophobic forces and van der Waals interaction forces. The higher drug loading capacity will provide, the higher intracellular concentration and higher killing efficiency in the tumor sites. The FNQ-MSN exhibited excellent stability in the PBS medium and the particle size remained unchanged throughout the study period until 30 days. Such improved stability the carrier system and high loading capacity of the carrier system will improve the biodistribution and efficiency in the tumor treatment.

### In Vitro Drug Release

**Fig. 2:**
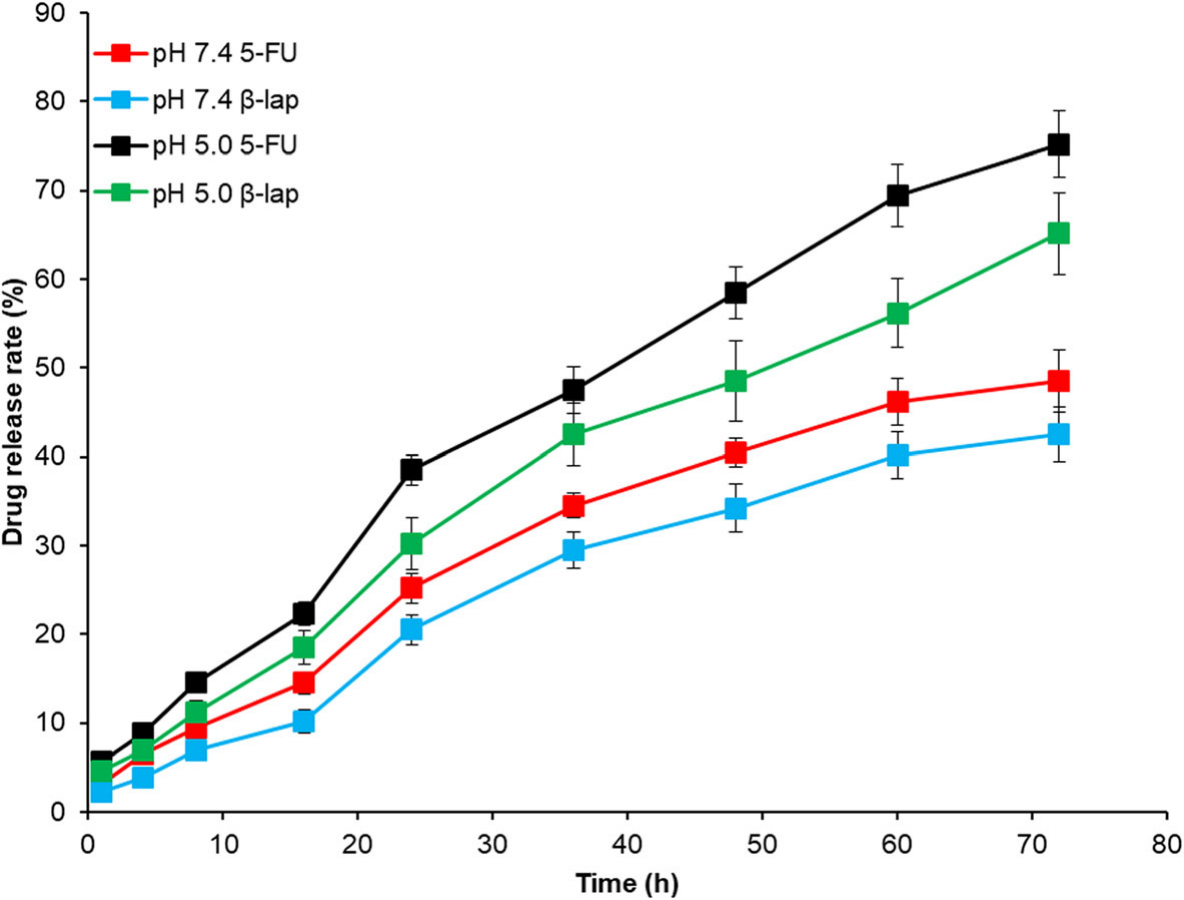
In vitro release of 5-FU and ß-lap from FNQ-MSN in pH 7.4 and pH 5.0 buffer conditions. The release study was continued until 72 h, and drug release was quantified using HPLC method. **p < 0.01

The in vitro drug release of 5-FU and ß-lap from FNQ-MSN was studied in PBS and ABS (Fig. 2). As shown, two different release trends were observed in pH 7.4 and pH 5.0. For example, 25% of 5-FU released in 24 h in pH 7.4 compared with ~ 40% of drug release in pH 5.0 in 24 h. Similarly, 20% and 30% of ß-lap was released in pH 7.4 and pH 5.0, respectively. The release kinetics was identical throughout the study period with 50% of 5-FU released in alkaline buffer and 80% of drug release at pH 5.0 after 72 h of study period. A pH-responsive release of drug in the acidic conditions is advantageous to the cancer treatment. No burst release phenomenon was observed in both the pH conditions mainly attributed to the presence of the DSPE-PEG layer that controlled the release of the drug from the FNQ-MSN throughout the study period. Significant difference in drug release was observed between pH 7.4 and pH 5.0 conditions. At pH 7.4 conditions, PEG-*b*-DSPE exhibits a high stealth layer property thereby preventing the release of the encapsulated compound. The present result showed that the drug release accelerated at pH 5.0, indicating the disassembly of the protective shell around the MSN surface. The pH-sensitive drug release rate, which ensures maximum drug release within the cancer cells, may improve antitumor efficacy and decrease unwanted toxicity to normal tissues. It is possible that in the presence of a pH-responsive element, FNQ-MSN might result in the shedding of lipid bilayer on the MSN resulting in enhanced release in the lower pH conditions.

### Sensitivity of NQO1 to ß-lap

**Fig. 3:**
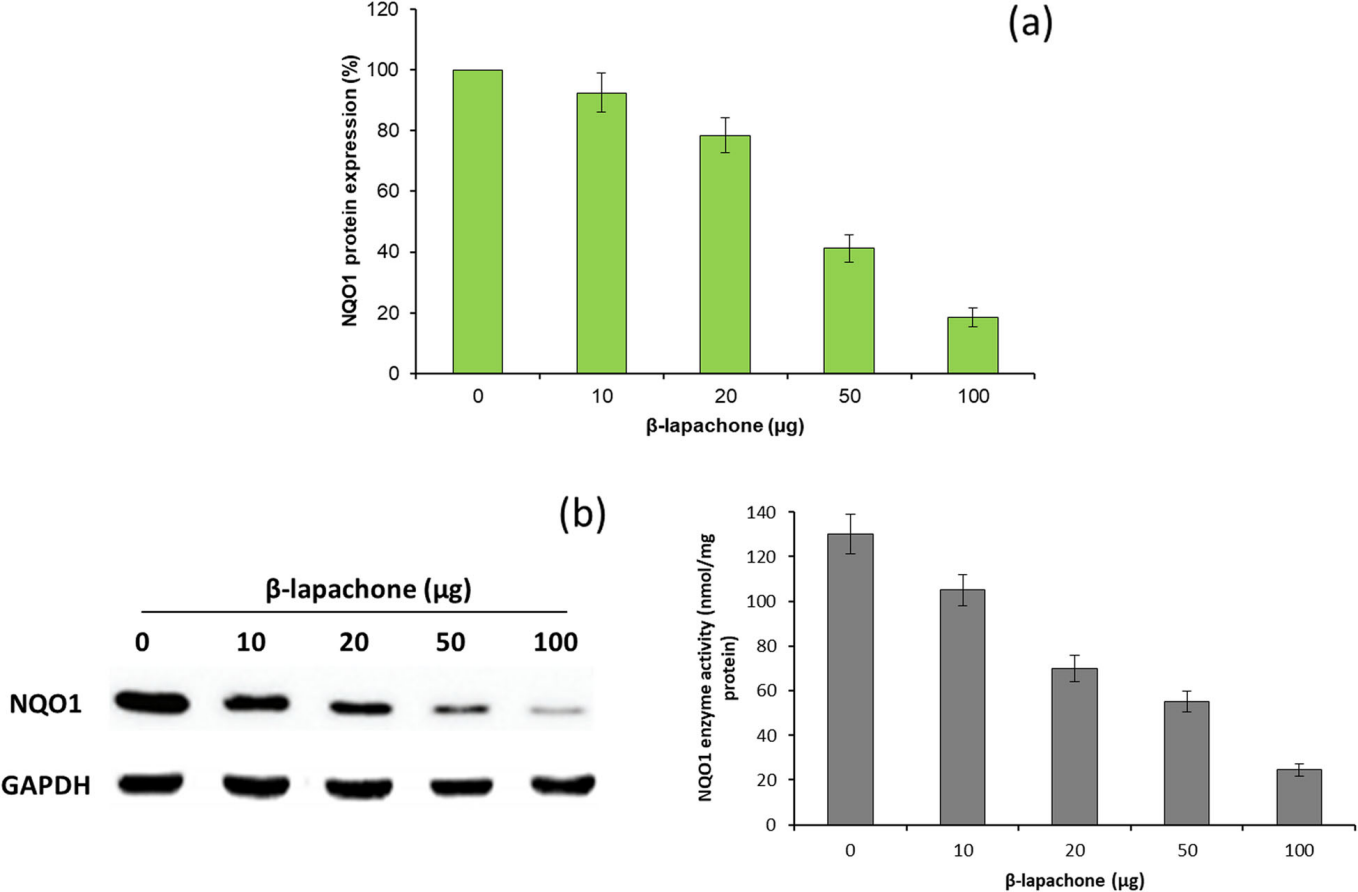
**a** Effect of concentration-dependent activity of ß-lap on the NQO1 activity of Cal33 cancer cells by the enzymatic method. **b** Effect of concentration-dependent activity of ß-lap on the NQO1 protein levels by western blot analysis using GAPDH as a housekeeping protein

As the upregulation of NQO1 in many cancer cells including HNSCC associated with poor prognosis and poor therapeutic outcome, drugs targeting NQO1 could be a potential strategy towards cancer treatment [24]. NQO1 is overexpressed in the level ranging from 100- to 200-fold in squamous cell carcinoma compared with that associated with normal tissues. Therefore, ß-lap influence on the NQO1 enzyme activity was studied in Cal33 cells. As shown, ß-lap showed a concentration-dependent inhibition of NQO1 enzyme activity in Cal33 cells (Fig. 3a). Notably, significant inhibitory effect was observed at a dose of 20–50 μg/ml of ß-lap. Furthermore, inhibitory potential of ß-lap on NQO1 protein was further studied by western blot analysis (Fig. 3b). Results clearly revealed the significant decrease in the NQO1 protein in the cancer cells with an increase in the concentration of ß-lap. Approximately, 80% NQO1 inhibition observed at 100 μg/ml of ß-lap. NQO1 bioactivatable drug such as ß-lap is metabolized by NQO1 and forms an unstable hydroquinone compound which immediately converts back to the original component leaving back 2 one-electron oxidations and consumes 2 O^2−^. This will create a futile redox cycle in which 1 molecule of ß-lap will generate 130 moles of superoxide at the cost of consuming 60–70 moles of NAD(P)H6. The so-formed superoxide (O2.−) radicals will get converted to hydrogen peroxide (H2O2−) and will cause cell apoptosis and cell death [25].

### In Vitro Anticancer Effect of FNQ-MSN in HNSCC Cells

**Fig. 4:**
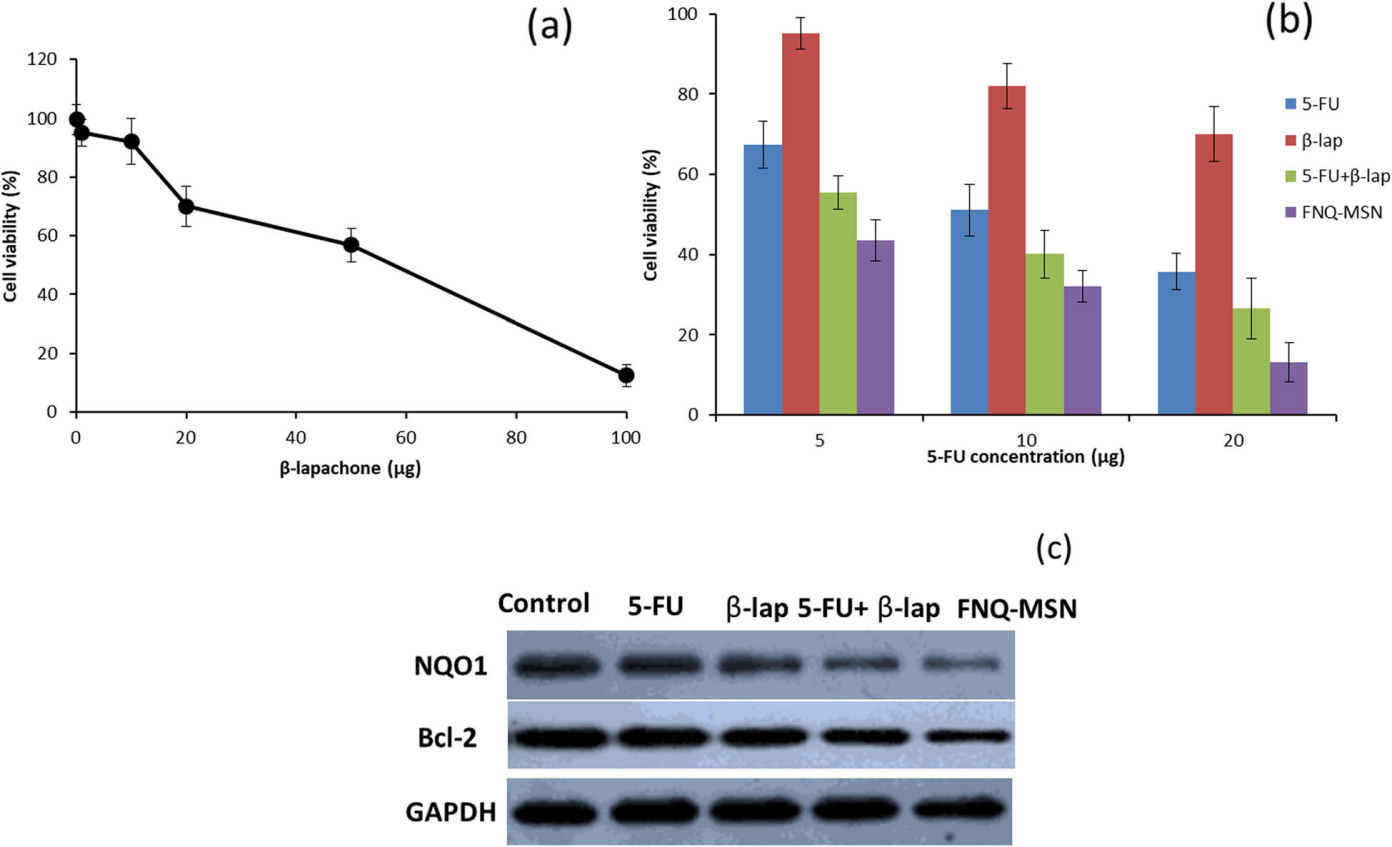
**a** Effect of concentration-dependent activity of ß-lap on the cell viability of Cal33 cells. **b** Cytotoxic effect of individual and combination with second chemotherapeutic agent, 5-FU at 3 different concentrations of 5–20 μg/ml, respectively. The cell viability was evaluated by MTT assay after 24 h of incubation. **c** Western blot analysis of NQO1 and Bcl-2 protein expression after treatment with individual and combination of 5-FU and ß-lap

The in vitro anticancer effect of ß-lap in Cal33 cell was studied by MTT assay. Results revealed that ß-lap exhibited a typical concentration-dependent decrease in cell viability (Fig. 4a). Notably, significant decrease in cell viability was observed at a concentration of 50 μg/ml. For further experiments, 20 μg/ml was selected (below the IC50 value of ß-lap). Next, potentiation effect of ß-lap on 5-FU was studied at 3 different concentrations (5, 10, and 20 μg/ml). As shown, combination of 5-FU+ß-lap resulted in lower cell viability; most notably, FNQ-MSN exhibited significantly lower cell viability compared with that of any of the individual drug or physical combinations (Fig. 4b). For example, 5-FU and ß-lap showed a cell viability of 35.7% and 70.2% while 5-FU+ß-lap showed 26.5% and FNQ-MSN showed 13.1%, respectively. Results clearly showed that the cytotoxic effect of 5-FU was significantly increased when combined with ß-lap. It is worth noting that FNQ-MSN was more effective compared with that of 5-FU+ß-lap attributed to the better internalization and controlled release of loaded therapeutics from the carrier system. Results clearly reveal the inhibitory effect of ß-lap on NQO1 enzymatic activity and protein levels and potentiated the anticancer effect of 5-FU and results in enhanced therapeutic outcome in the treatment of HNSCC.

### FNQ-MSN Role in the Signaling Pathways: Western Blot Analysis

The mechanism of the ß-lap-mediated cytotoxic effect was further explored by western blot analysis. As shown, ß-lap resulted in a decrease in the protein band of NQO1 compared with control; however, most notable decrease in the NQO1 level was observed in FNQ-MSN-treated cell group (Fig. 4c). The ß-lap-induced enhancement of the chemosensitivity of Cal33 by enhancing the cell apoptosis was evaluated through the Bcl-2 protein level. The Bcl-2 family plays an important role in regulating the cell homeostasis and controls the proliferation and death of cancer cells. The process of decrease of Bcl-2 and increase of Bax results in releasing the cytochrome C in the cytosol that will initiate the entire caspase cascade resulting in cell death [26]. Results clearly highlighted that the combination of 5-FU and ß-lap (FNQ-MSN) significantly downregulated the Bcl-2 indicating the enhanced anticancer effect in the Cal33 cancer cells.

### Apoptosis Analysis of HNSCC

**Fig. 5:**
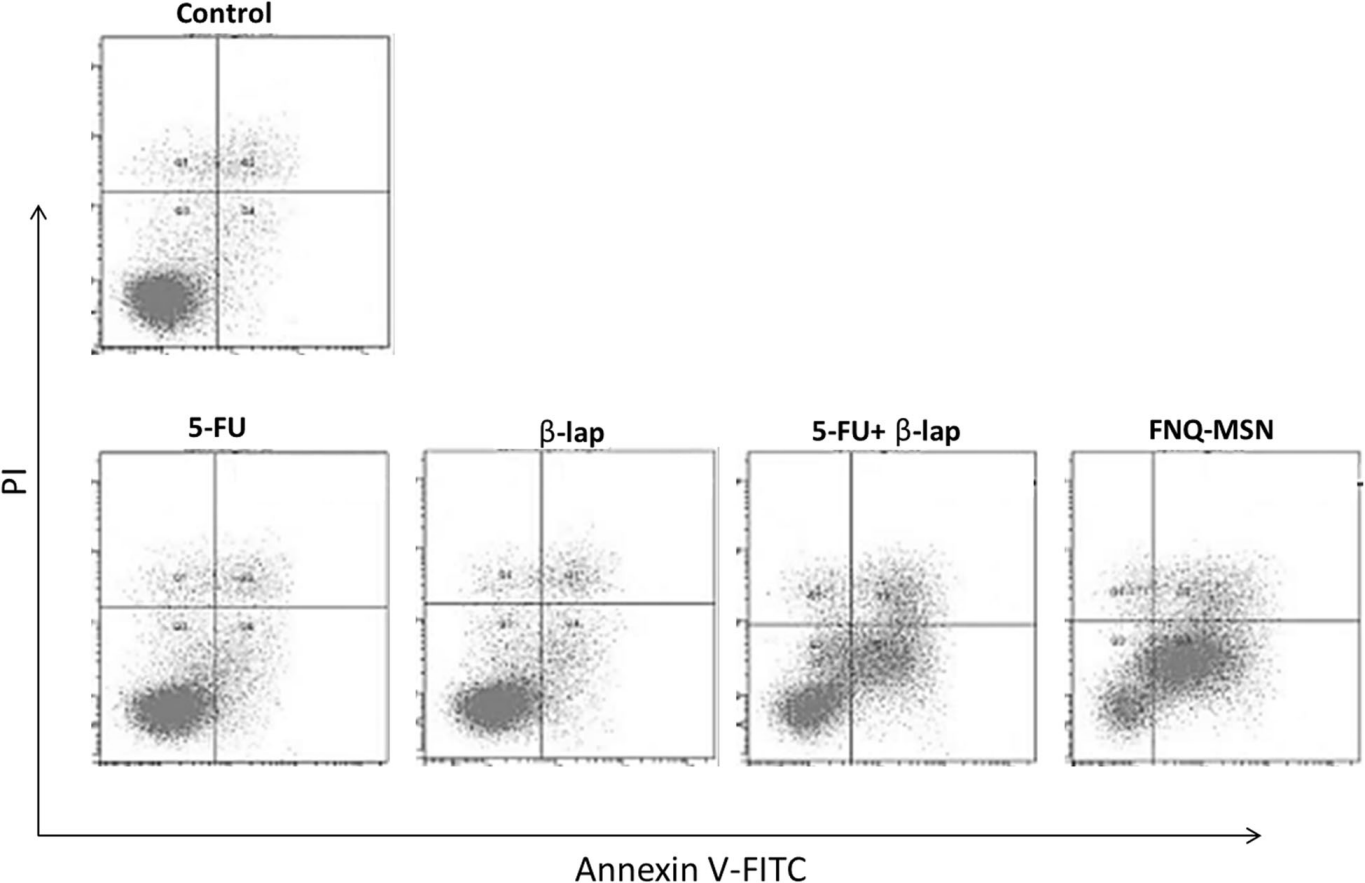
Flow cytometer analysis of Cal33 cells using double staining of annexin V and PI after treatment with individual and combination of 5-FU and ß-lap. 10,000 events were recorded in the flow cytometer

**Fig. 6:**
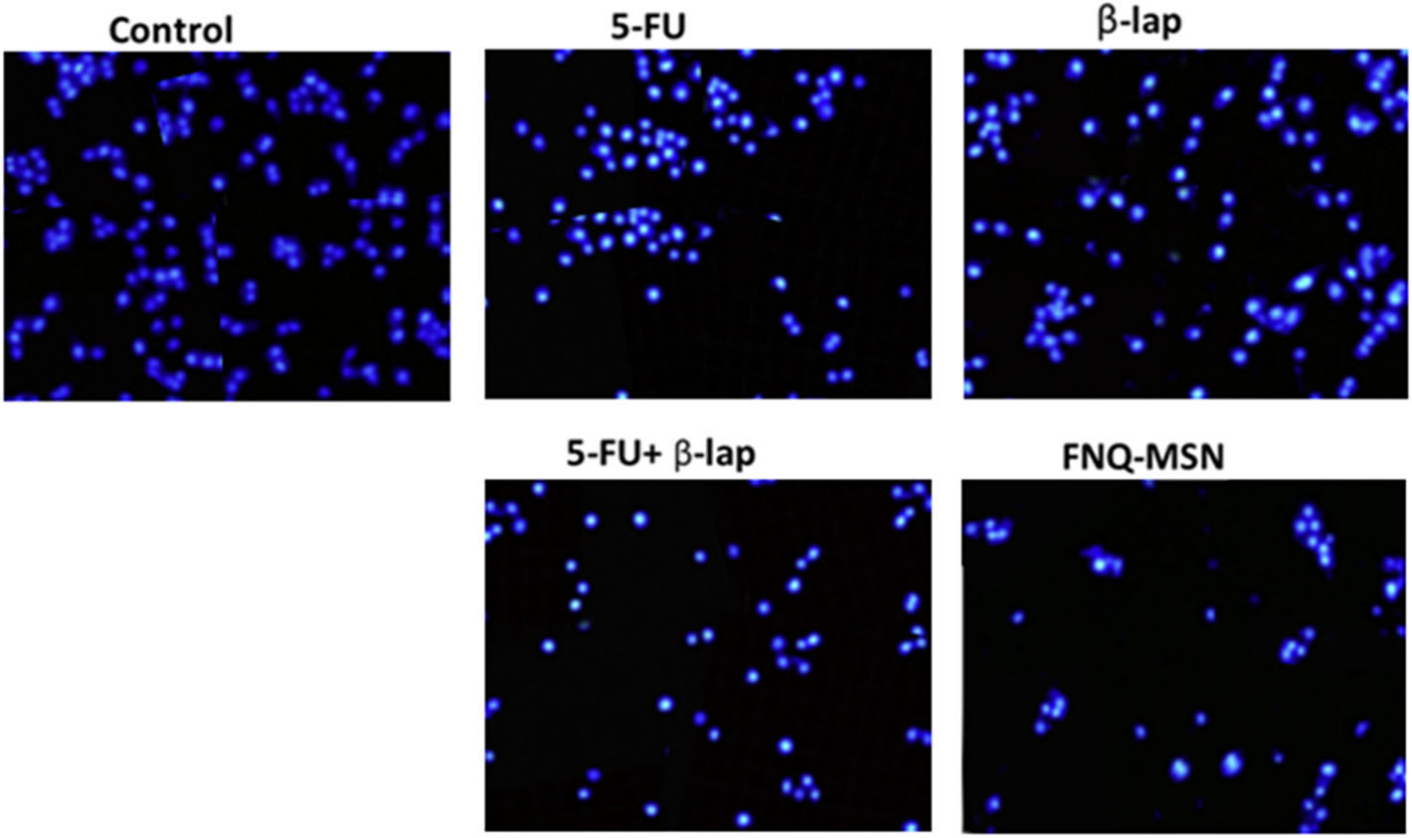
Nuclear morphology analysis of Cal33 cells after staining with Hoechst 33342; cells were treated with individual and combination of 5-FU and ß-lap. 10,000 events were recorded in the flow cytometer

Following the cell viability analysis, apoptosis effect of individual and combined drug on Cal33 cells was evaluated by flow cytometer after annexin V/PI staining (Fig. 5). The apoptosis effect of individual 5-FU (10 μg/ml) or ß-lap (30 μg/ml) did not result in any appreciable apoptosis of cancer cells; however, 5-FU+ß-lap resulted in a dramatic increase in the apoptosis proportion of cancer cells. Importantly, FNQ-MSN resulted in more than 60% of cell apoptosis (early and late apoptosis) signifying the superior therapeutic effect of carrier-based combination regimen. Apoptosis effect was further confirmed by Hoechst 33342 staining. As shown, cells treated with FNQ-MSN results in a higher amount of cells undergoing apoptosis, nuclear condensation, and fragmentation features compared with that of either 5-FU or ß-lap alone (Fig. 6). During the apoptosis process, cells shrink without being damaged in the outer cell membrane and results in chromatin condensation with increased apoptotic body formation.

### Live/Dead Assay

**Fig. 7:**
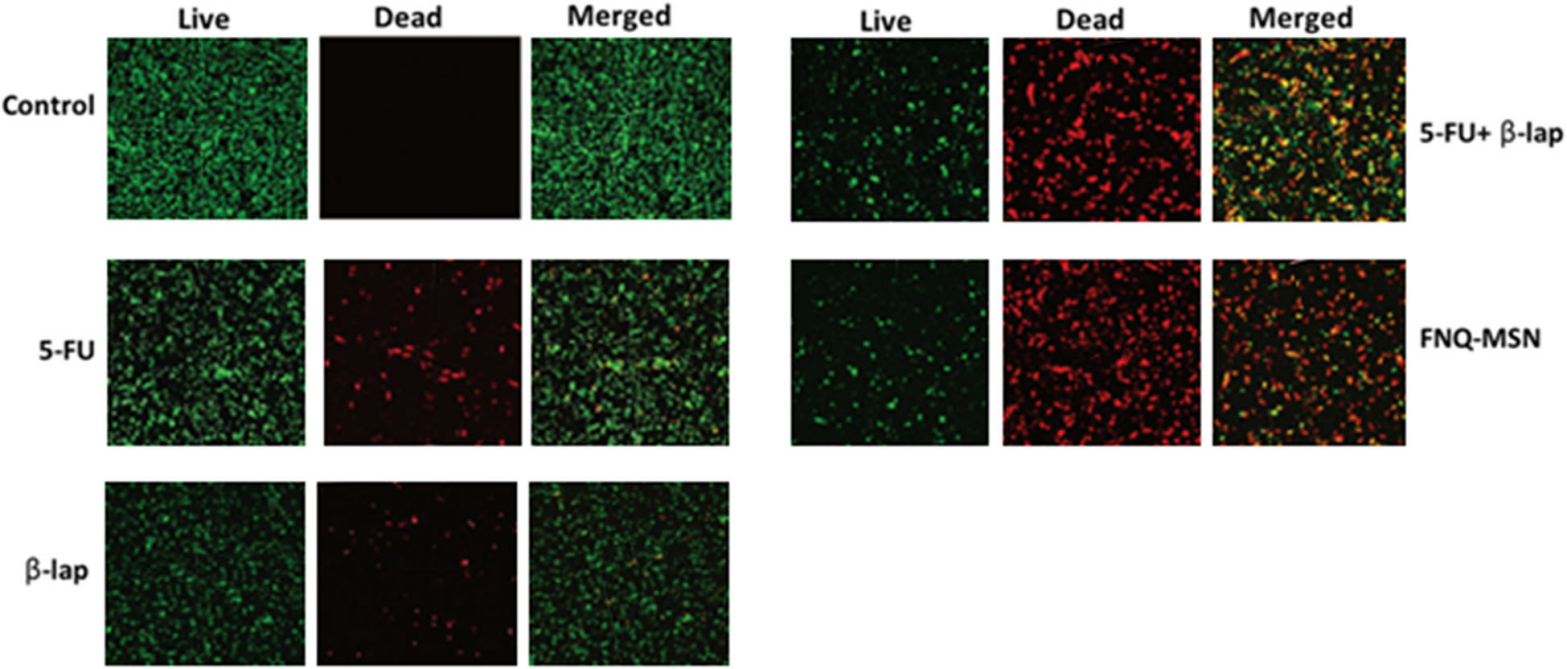
Live/dead analysis of Cal33 cells using double staining of Calcein AM and ethidium bromide after treatment with individual and combination of 5-FU and ß-lap

The anticancer effect of individual and combined drug was further evaluated by the Live/Dead assay. The treated cells were subjected to Calcein AM and ethidium bromide staining as a respective marker of live and dead cells (Fig. 7). As shown, untreated cells are stained with 100% of green fluorescence. The individual drugs, 5-FU (10 μg/ml) or ß-lap (30 μg/ml) though showed slight red fluorescence stained cells; however, predominant cells exhibited green fluorescence indicating limited cell death. Remarkable cell death was observed in FNQ-MSN-treated cancer cells as shown by predominant red fluorescence and fewer green fluorescence stained cells. The higher anticancer effect of FNQ-MSN was attributed to the synergistic activity of 5-FU and ß-lap in the cancer cells and the ability of ß-lap to increase the chemosensitivity of 5-FU.

### In Vivo Antitumor Efficacy of FNQ-MSN Against HNSCC Xenograft Model

**Fig. 8:**
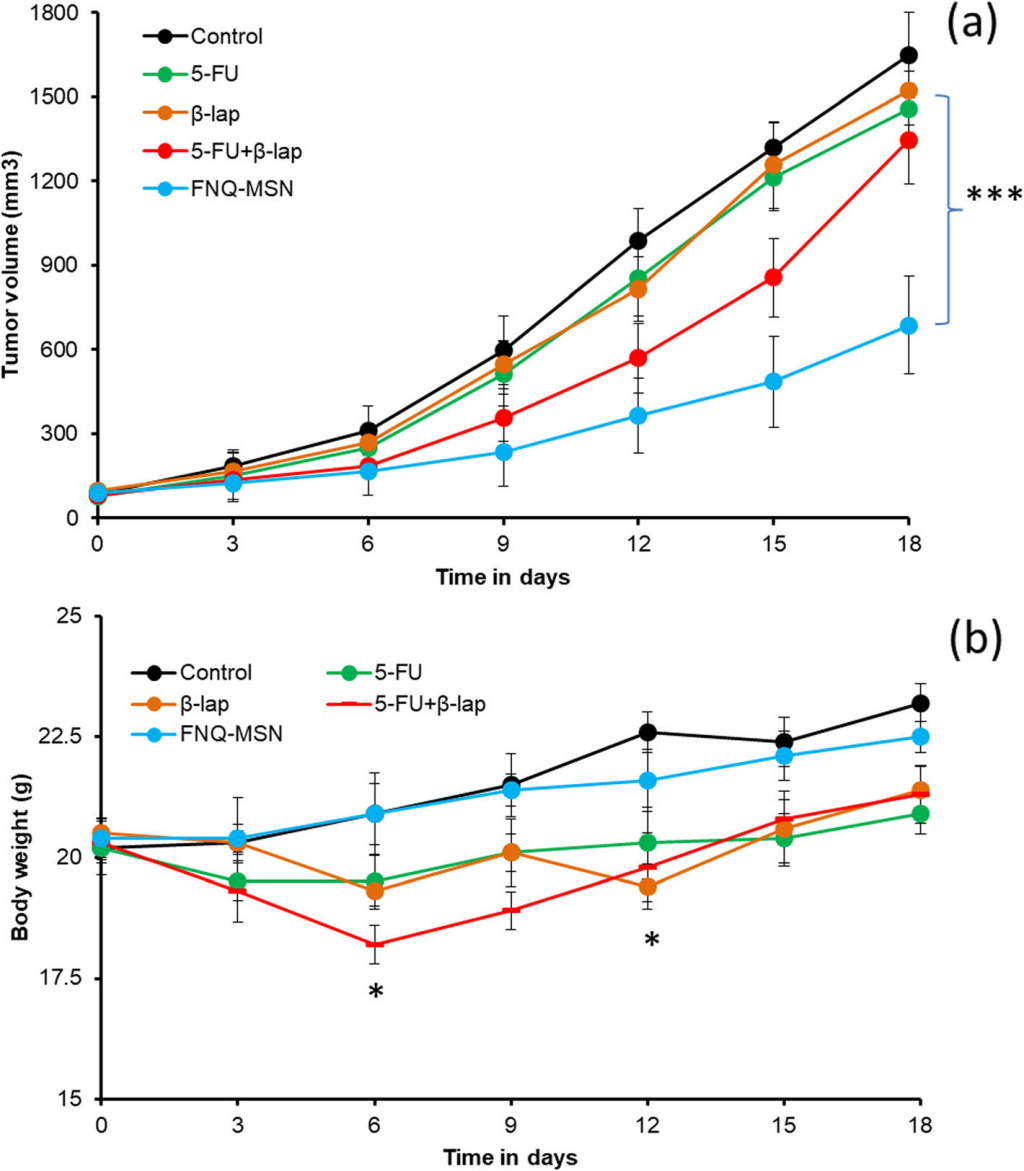
**a** In vivo antitumor efficacy of FNQ-MSN in HNSCC xenograft model. The mice bearing HNSCC tumors were treated with 5-FU, ß-lap, 5-FU+ß-lap and FNQ-MSN at a fixed dose of 5 mg/kg of 5-FU and 10 mg/kg of ß-lap intravenously for 3 times. Tumor volume was noted as a part of efficacy analysis and compared with non-treated control. **b** Body weight analysis corresponding to tumor volume data. *p < 0.05 and ***p < 0.001

To further evaluate the efficacy of combinational regimen in an animal model, HNSCC xenograft was prepared and administered with 5 mg/kg of 5-FU and 10 mg/kg of ß-lap, respectively. The pharmaceutical grade of ß-lap is ARQ761 which is presently in phase I clinical trial for the treatment of metastatic malignancies. ß-lap has set the limit for the maximum tolerable dose in humans as per the preclinical studies [27]. Keeping this in mind, we have optimally selected a dose of 10 mg/kg of ß-lap for the in vivo studies. As shown, no significant decrease in tumor volume was observed with individually administered 5-FU and ß-lap compared with that of non-treated control (Fig. 8a). Notable decrease in tumor volume was observed with a physical mixture of 5-FU+ß-lap; however, combined treatment of carrier-based 5-FU and ß-lap (FNQ-MSN) significantly delayed the tumor growth and prolonged the survival of tumor-bearing xenograft mice. The tumor volume graph of FNQ-MSN could be divided into two parts; insignificant growth in tumor volume was observed until day 9 while tumor significantly increased after day 9 until day 18, nevertheless, overall tumor volume was manifold smaller compared with that of control or other groups. The toxicity concern of the individual and combinational regimen was evaluated in terms of body weight (Fig. 8b). As shown, physical mixture of 5-FU+ß-lap resulted in severe toxicity as represented by more than 10% loss in body weight while 5-FU resulted in 5% of body weight. As expected, FNQ-MSN did not result in any loss of body weight indicating the unique advantage of the lipid-MSN-based carrier system. The enhanced antitumor effect of FNQ-MSN was attributed to the inhibitory effect of ß-lap towards the NQO1 enzyme and protein which in turn increased the chemosensitivity of 5-FU and resulted in synergistic anticancer effect in the Cal33 human tumors, second, lipid bilayer–coated MSN protected the drug during the systemic circulation and might release in a controlled manner in the tumor tissue, third, PEGylation of nanocarrier might prolonged the blood circulation time allowing for the preferential accumulation in the tumor tissues owing to EPR effect [28, 29]. PEGylated MSN were mainly trapped in RES of the liver, spleen, and lung, accounting for over 80% of the administered dose. PEG modification of MSN obviously decreased the clearance rate of MSN in organs, demonstrating that the PEGylated NPs were more difficult to be removed from the RES organs regardless of the particle shape [30, 31]. Overall, in vivo study on HNSCC model offer a “proof of concept” that effective combination of 5-FU+ß-lap in stable nanocarrier might enhance the therapeutic efficacy in tumor while alleviating the associated toxic effect.

## Data Availability

All data generated or analyzed during this study are included in this published article and its supplementary information files.

## Abbreviations

5-FU: 5-Fluorouracil
EPR: Enhanced permeation and retention effect
FNQ-MSN: 5-FU/ß-lap-loaded mesoporous silica nanoparticles
HNSCC: Head and neck squamous cell carcinomas
MSN: Mesoporous silica nanoparticle
NQO1: NAD(P)H:quinone oxidoreductase 1
ß-lap: ß-lapachone
